# Ontology-Based Meta-Analysis of Animal and Human Adverse Events Associated With Licensed Brucellosis Vaccines

**DOI:** 10.3389/fphar.2018.00503

**Published:** 2018-05-15

**Authors:** Jiangan Xie, Jessica Wang, Zhangyong Li, Wei Wang, Yu Pang, Yongqun He

**Affiliations:** ^1^School of Bioinformatics, Chongqing University of Posts and Telecommunications, Chongqing, China; ^2^Department of Computational Medicine and Bioinformatics, University of Michigan Medical School, Ann Arbor, MI, United States

**Keywords:** *Brucella*, brucellosis vaccine, adverse event, abortion AE, Ontology of Adverse Events, ANOVA statistics analysis

## Abstract

*Brucella abortus* strain 19 (S19), *Brucella melitensis* Rev 1 (Rev1), and *B. abortus* strain RB51 (RB51) are the three licensed animal brucellosis vaccines, and they have been most commonly and successfully used in prevent brucellosis in animals. However, many adverse events (AEs) have been associated with these three vaccines after their administering to animals or being accidentally exposed to humans. In this study, 27 peer-reviewed publications containing animal and human AE reports associated with these three brucellosis vaccines were manually annotated from the PubMed database. Our meta-analysis identified 20 animal AEs and 46 human AEs associated with the three vaccines. Based on the Ontology of Adverse Events (OAE) hierarchical classification, these animal AEs were enriched in the immune and reproductive systems that might eventually result in the occurrence of abortion or infertility. The human AEs were concentrated in the behavioral and neurological conditions, and these AEs showed flu-like symptoms that are consistent with human brucellosis. Furthermore, an analysis of variance (ANOVA) statistics analysis with linear model fits was used to determine the major variables that might affect the occurrence of abortion AE in animals. The ANOVA results indicated that three variables (*P*-value < 0.05) are significantly associated with the occurrence of abortion AE: animal species, vaccination dose, and vaccination route. The other two variables (i.e., vaccine type and animal age at vaccination) did not significantly (*P*-value > 0.05) associated with the occurrence of abortion AE. Overall, this study represents the first ontology-based meta-analysis of adverse events associated with animal vaccines. The results of such a study led to the better understanding of brucellosis vaccine AEs, facilitating rational design of more secure and effective vaccines.

## Introduction

Brucellosis, the most common bacterial zoonosis worldwide, is caused by Gram-negative intracellular coccobacillus *Brucella* (Corbel, 1997). To date, the brucellosis remains a significant threat to animals and humans, especially in many developing areas of the world (Nicoletti, 2010). *Brucella* spp. infects a variety of domestic and wild animals such as cattle, sheep, goats, pigs, and dogs. *Brucella* infections of these animals may result in serious illnesses (e.g., arthropathy and abortion) and even death. Humans are infected with *Brucella* usually through contact with infected animals or contaminated animal products (e.g., milk and flesh). As reported in 2006, an annual average of 110 human *Brucella* infection cases were reported to the National Notifiable Diseases Surveillance System (NNDSS) in the United States; however, more than half a million of new human brucellosis cases occurred each year worldwide (Pappas et al., 2006). In humans, undulant fever is the most common symptom of brucellosis, followed by osteoarticular involvement (e.g., arthralgia and arthritis), sweating, and constitutional symptoms (e.g., malaise and weight loss) (Franco et al., 2007).

Vaccination has significantly contributed to the prevention and eradication of brucellosis worldwide. Many developed countries have successfully launched brucellosis eradication programs. For example, the US State-Federal Brucellosis Eradication Program was established in 1934 as part of an economic recovery program to eliminate brucellosis in cattle. Owing to the combinatorial programs of vaccination and slaughtering of brucellosis-positive cattle, this program has had considerable success for there were no brucellosis affected cattle herds as of 30 November 2001 in the United States (Ragan, 2002), and all US states remain classified as Class Free for bovine brucellosis in 2013 (USAHA, 2017). Undoubtedly, vaccination is one of the most effective measures toward the success of the brucellosis eradication program. In the past several decades, three vaccines have been most widely used to prevent brucellosis, i.e., *Brucella abortus* strain 19 (S19), *Brucella melitensis* Rev 1 (Rev1), and *B. abortus* strain RB51 (RB51) (Schurig et al., 2002). The S19 and Rev1 have been successfully used in many developed countries to control bovine brucellosis, but both vaccines can induce antibodies to *B. abortus* lipopolysaccharide (LPS) O-side chain that are used in brucellosis serologic diagnosis. The presence of the antibodies result in the difficulty in differentiation between infected and vaccinated animals (Gonzalez et al., 2008). The RB51 is a rifampicin-resistant rough mutant of *B. abortus* strain 2308 that lacks most of the antigenic LPS O-side chain, and it does not elicit positive responses on brucellosis serologic diagnosis tests. All these three vaccines cannot be used for humans due to their virulence and infections on humans. To date, there has not been safe and effective licensed vaccine for prevention of human brucellosis.

Although these existing vaccines have been successfully used in preventing brucellosis in animals, adverse events (AEs) often occur after vaccination. For example, abortion is the most commonly AE associated with S19 and Rev1 when they were vaccinated to pregnant cows (Schurig et al., 2002). Additionally, humans, especially those people with special occupations (e.g., veterinarians, veterinary technicians, and ranch employees) may be inadvertently exposed to these vaccines by means of unintentional inoculation or other routes of exposure (e.g., experimental vaccination). Many AEs in humans (e.g., fever, headache, and sweats) have been reported to be associated with the accidental exposure to the livestock brucellosis vaccines (Spink et al., 1962; Ollé-Goig and Canela-Soler, 1987; Ashford et al., 2004).

The existing researches have provided various reports of AEs associated with the brucellosis vaccines S19, Rev 1, and/or RB51, however, a systematic comparative study on the AEs associated with these vaccines used for animals or unintentionally inoculated to humans has not been reported. In this study, we focus on a meta-analysis of AEs associated with these three commonly used brucellosis vaccines in animals and humans. To collect the reported AEs, all of the available data reported in articles published in PubMed database were annotated. These reported AEs were represented and classified using the Ontology of Adverse Events (OAE) (He et al., 2014). Furthermore, we performed a detailed statistical analysis of each variable in the process of vaccination using the analysis of variance (ANOVA) method with linear model fits (Todd et al., 2013) to ascertain which variables will affect the abortion AE outcome in animals. This is the first ontology-based study to meta-analyze the AEs associated with the three licensed brucellosis vaccines in vaccinated animals as well as in unintentionally inoculated humans.

## Materials and Methods

**FIGURE 1:**
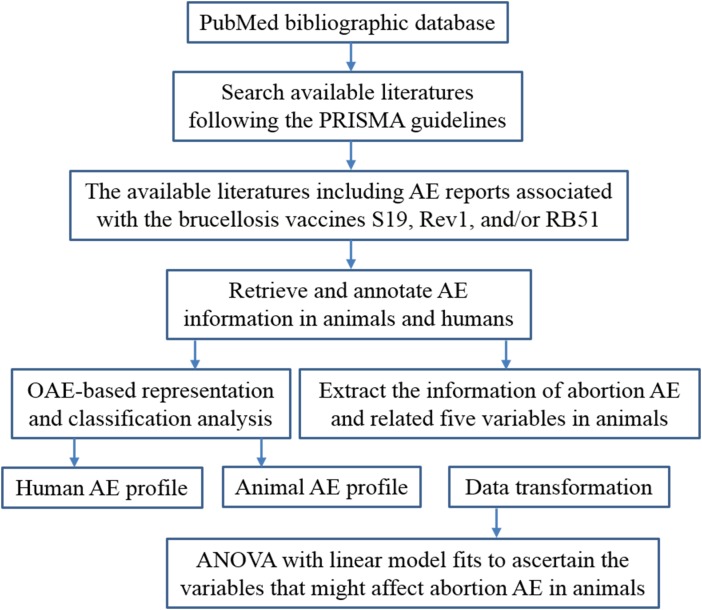
Overall project workflow.

The general project workflow shown in **Figure 1** outlines different method steps in this study. The details of these research processes are provided below.

### Extract AE Data From PubMed Literatures

A meta-analysis of previous written studies on the AEs related to brucellosis vaccines was performed by following the PRISMA (Preferred Reporting Items for Systematic Reviews and Meta-Analyses) guidelines (Moher et al., 2015). Briefly, the meta-analysis was done by searching the PubMed bibliographic database^1^ with the search details (“brucella” [MeSH Terms] OR “brucella” [All Fields]) AND (“vaccines”[MeSH Terms] OR “vaccines” [All Fields] OR “vaccine” [All Fields]). The abstracts and full texts of those papers reporting AEs associated with brucellosis vaccines S19, Rev1, and/or RB51 were retrieved and annotated independently by two reviewers (the authors JX and JW). Any disagreements of the annotated results were resolved by discussions and analysis with a third reviewer (the corresponding author YH). From each eligible paper, the information of AEs and related five variables (i.e., animal species, vaccine type, vaccination dose, vaccination route, and animal age at vaccination) during brucellosis vaccination were identified and recorded in a Microsoft Excel.

### OAE-Based AE Representation and Classification

The OAE is a community-driven ontology developed to standardize and integrate data relating to AEs arising subsequently to medical interventions (e.g., medication and vaccination), as well as to support computer-assisted reasoning (He et al., 2014). Previous studies have shown that OAE performed well in the analysis of AE data associated with vaccines and drugs (Sarntivijai et al., 2012, 2016; Xie et al., 2016a,b). In this study, the OAE was used for representing and classifying the AEs associated with the three brucellosis vaccines in animals and humans. For OAE classification, the AEs associated with these three brucellosis vaccines and the AE-related parent term hierarchies were extracted from OAE using the OntoFox program (Xiang et al., 2010), and the hierarchical structures of these terms were visualized using the Protégé-OWL editor^2^.

### Data Transformation

Five major variables include animal species, vaccine type, vaccination dose, vaccination route, and animal age at vaccination that identified in the process of brucellosis vaccination were selected to test which variables have a strong impact on the occurrence of abortion AE. Notably, human was not included in the species in this statistical analysis for abortion AE. The variables animal age at vaccination and vaccination dose use the continuous values. Specifically, the animal age adopts the unit of year, and log10 transformation was applied for the value of vaccination dose. The raw data of other three variables (i.e., vaccine type, vaccination route, and animal species) was transformed to discretized data using a data discretization process. For example, the variable “vaccination route” has four values: subcutaneous vaccination, conjunctival vaccination, oral vaccination, and intravenous vaccination. During the data discretization step, the number string values of these four vaccination routes were discretized to four discrete digital values 1, 2, 3, and 4, respectively.

### Statistical Analysis of Variables that Significantly Affected Abortion AE

The ANOVA was first used to analyze which major variables in the process of vaccination with these three brucellosis vaccines contributed significantly to the occurrence of abortion. In ANOVA statistics analysis, the abortion AE was set as a dependent variable while the other five variables were set as independent variables. The ANOVA output is a *P*-value data set that corresponds to a list of *P*-values for different independent variables. The statistical results indicate how each variable affected the abortion occurrence.

In this study, we use *R* program to perform the statistics analysis of ANOVA with linear model fits (**Supplementary Presentation S1**). The version R 3.3.3 was freely downloaded from website^3^ (Accessed at May 01, 2017). In ANOVA for linear model fits, specifying a single object gives a sequential analysis of variance table for that fit. That is, the reductions in the residual sum of squares as each term of the formula is added in turn are given in as the rows of a table, plus the residual sum of squares. The table will contain F statistics (and *P*-values) comparing the mean square for the row to the residual mean square.

## Results

### Literature Meta-Analysis of AEs Associated With the Three Brucellosis Vaccines

**FIGURE 2:**
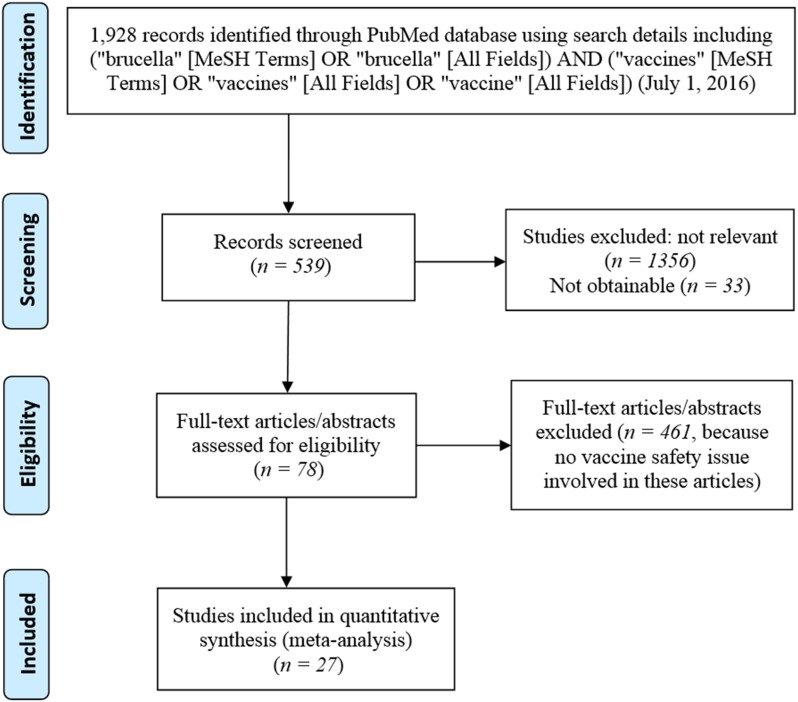
Preferred Reporting Items for Systematic Reviews and Meta-Analyses (PRISMA) diagram of records identification and selection.

**Figure 2** is the PRISMA flowchart of the meta-analysis of brucellosis vaccine AE-related papers in this study. At last, a total of 27 papers (**Supplementary Presentation S2**) were identified to have reported the AEs associated with at least one of these three vaccines. Specifically, six of these 27 papers (22.2%) reported AEs associated with S19 vaccination, 7/27 (25.9%) papers reported AEs associated with Rev1 vaccination, and 16/27 (59.3%) papers reported AEs associated with RB51 vaccination.

**FIGURE 3:**
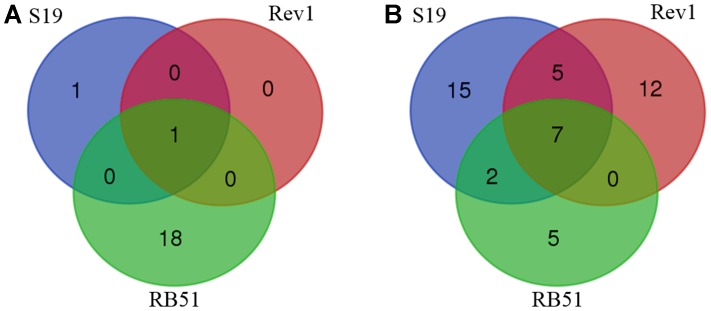
Venn diagram summary of adverse events (AEs) associated with S19, Rev1, and RB51. **(A)** Animal AEs associated with the three vaccines. **(B)** Human AEs associated with the three vaccines.

Our meta-analysis identified a total of 20 AEs associated with S19, Rev1, and/or RB51 in 15 different species of animals (**Table 1**). Among the 20 AEs (**Table 1** and **Figure 3A**), abortion AE is the only one that has been reported to the vaccination with Rev1. Two AEs including arthropathy and abortion were reported to be associated with S19. For RB51, 19 AEs were reported. Furthermore, the abortion was the only AE shared in all the three vaccines, and no AE was shared between any two vaccines (**Figure 3A**). Due to accidental vaccine exposures in humans, 29, 24, and 14 human AEs were reported to be associated with S19, Rev1, and RB51, respectively (**Figure 3B** and **Table 2**). Based on the Venn diagram analysis (**Figure 3B**), 7 AE symptoms were shared in all the three vaccines (i.e., redness, sweating, fever, swelling, chills, headache, and hospitalization). Specifically, S19 and Rev1 shared five AE symptoms (i.e., conjunctivitis, asthenia, acute brucellosis, soreness, and malaise); S19 and RB51 shared two AE symptoms (i.e., myalgia and arthralgia). In total, 46 unique AEs were reported to be associated with S19, Rev1, and/or RB51 in humans (**Figure 3B**).

**Table 1:** The adverse events associated with S19, Rev1, and/or RB51 in animals.

Adverse event	Vaccine and animal
**Hepatobiliary AE (1)**
Liver infiltration	RB51: deer mice
**Immune system AE (11)**
Disseminated histiocytic pneumonia	RB51: deer mice
Endometritis	RB51: bison
Lymphoplasmacytic myocarditis	RB51: vole
Metritis	RB51: deer mice
Mycotic pneumonia	RB51: raven
Myocarditis	RB51: deer mice
Peritonsillar abscess	RB51: bear
Placentitis	RB51: beagle and bison
Pneumonia	RB51: deer
Purulent abscess	RB51: buffalo
Serositis	RB51: raven
**Infection AE (2)**
Intestinal helminthiasis	RB51: deer mice
Tetanus	RB51: buffalo
**Musculoskeletal AE (1)**
Arthropathy	S19: calf
**Prenatal AE (2)**
Abortion	S19: cow; Rev1: ewe, goat, and sheep; RB51: bison
Dystocia	RB51: heifer
**Reproductive system AE (1)**
Lymphoplasmacytic epididymitis	RB51: bull elk
**Serious AE (1)**
Death	RB51: deer and deer mice
**Syndrome AE (1)**
Cachexia	RB51: buffalo

*The top-level categories follow the Ontology of Adverse Events (OAE) hierarchy. AEs, adverse events.*

**Table 2:** The adverse events associated with S19, Rev1, and/or RB51 in humans.

Adverse event	Vaccine
**Behavioral and neurological AE (14)**
Abdominal pain	Rev1
Arm pain	Rev1
Bback pain	Rev1
Body ache	Rev1
Chills	S19, Rev1, and RB51
Depression	S19
Fatigue	RB51
Headache	S19, Rev1, and RB51
Insomnia	S19


Irritability	S19


Joint pain	Rev1


Leg pain	S19


Malaise	S19 and Rev1
Myalgia	S19 and RB51
**Digestive system AE (4)**
Diarrhea	S19 and RB51


Dyspepsia	S19


Nausea	S19
Vomiting	RB51
**Gustatory system AE (1)**
Anorexia	Rev1
**Hematopoietic system AE (1)**
Splenomegaly	S19
**Hepatobiliary AE (1)**
Hepatic lesion	S19
**Homeostasis AE (1)**
Fever	S19, Rev1, and RB51
**Immune system AE (9)**
Conjunctivitis	S19 and Rev1
Lymphadenopathy	S19
Lymphangitis	Rev1
Lymphocytosis	S19
Pyelonephritis	Rev1
Rectal abscess	S19


Spleen disorder	Rev1


Supraclavicular lymphadenopathy	Rev1


Tonsillitis	S19
**Infection AE (1)**
Acute brucellosis	S19 and Rev1
**Investigation result abnormal AE (1)**
Weight loss	S19
**Local AE (3)**
Redness	S19, Rev1, and RB51
Soreness	S19, Rev1
Swelling	S19, Rev1, and RB51
**Musculoskeletal AE (3)**
Arthralgia	S19 and RB51
Asthenia	S19 and Rev1


Muscle rigidity	Rev1
**Respiratory system AE (1)**
Sore throat	Rev1
**Reproductive system AE (1)**
Sexual dysfunction	S19
**Serious AE (1)**
Hospitalization	S19, Rev1, and RB51
**Skin AE (3)**
Erythema	RB51
Induration	RB51


Sweating	S19, Rev1, and RB51

*The top-level categories follow the OAE hierarchy*.

### OAE-Based Hierarchical Analysis of Brucellosis Vaccine AEs in Animals and Humans

**FIGURE 4:**
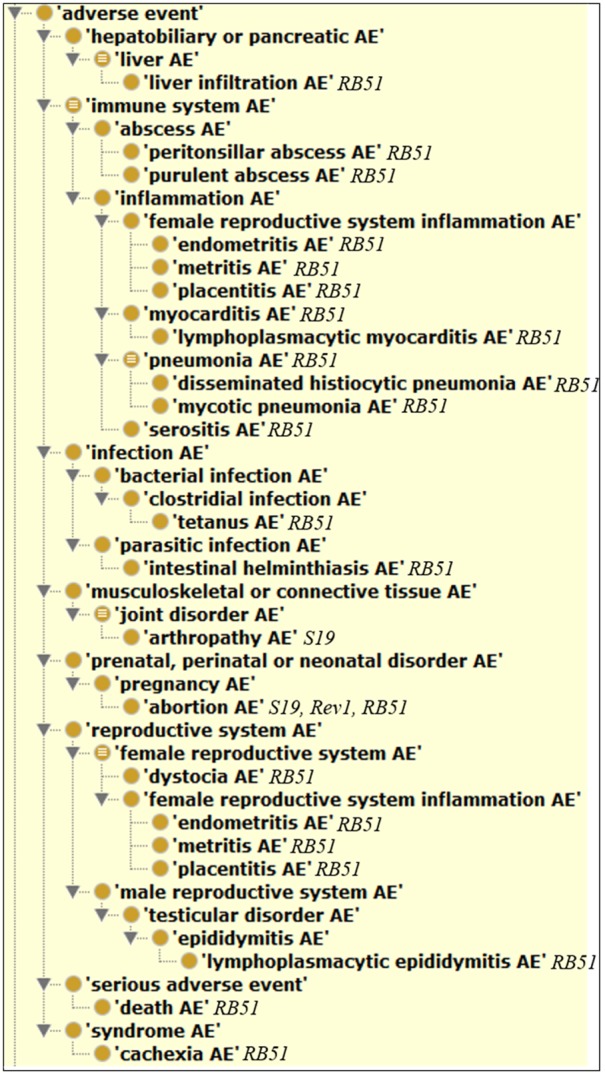
Classification of 20 animal AEs associated with S19, Rev1, and/or RB51 using Ontology of Adverse Events (OAE).

**Figure 4** shows the hierarchical classification of 20 animal AEs associated with S19, Rev1, and/or RB51 using the OAE inferred hierarchy structure. The most detailed animal AE diagnostic category is the ‘immune system AE’ (**Figure 4**), which includes 11 unique AEs symptoms (**Table 1**). Out of the 11 immune system AEs, three of them (i.e., endometritis, metritis, and placentitis) belong to the subclass ‘female reproductive system inflammation AE’ that all occurred in the reproductive system (**Figure 4**). These AEs may eventually result in the occurrence of abortion or infertility.

**FIGURE 5:**
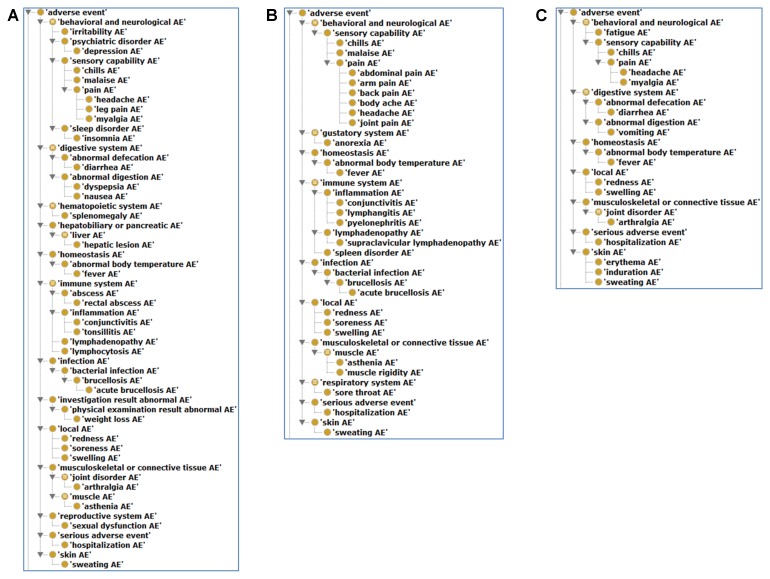
Classification of human AEs associated with S19, Rev1, and RB51 using OAE. **(A)** The hierarchy of S19-associated human AEs. **(B)** The hierarchy of Rev1-associated human AEs. **(C)** The hierarchy of RB51-associated human AEs.

As shown in **Figure 5**, the most detailed human AE diagnostic category associated with S19, Rev1, and RB51 is the behavioral and neurological AE, especially its subclass pain AE, which includes seven unique AE symptoms (i.e., headache, leg pain, abdominal pain, arm pain, body ache, joint pain, and myalgia). Additionally, these three vaccines were all associated with local AEs (i.e., redness, soreness, and swelling). A local AE was defined as erythema or induration of any size or duration at the site of injection or splash (Ashford et al., 2004). Specifically, for S19 and Rev1 (**Figures 5A,B**), the AEs included in immune system (e.g., conjunctivitis, lymphangitis, and lymphadenopathy) were also comparatively frequently reported AEs. Note that we have not yet found any immune system AE associated with RB51 in humans (**Figure 5C**).

### ANOVA Statistics Analysis Identified Variables Contributing Significantly to the Occurrence of Abortion AE in Animals

The original data curated from the 27 peer-reviews articles and the results of data transformation are provided in **Supplementary Data Sheet S1**. **Table 3** summarizes all of scoring parameters for the independent variables that resulted from ANOVA statistics analysis. The ANOVA results indicated that three variables (i.e., animal species, vaccination dose, and vaccination route) are significantly associated with the occurrence of abortion AE (*P*-value < 0.05). The other two variables (i.e., vaccine type and animal age at vaccination) did not significantly associated with the occurrence of abortion AE (*P*-value > 0.05).

**Table 3:** Analysis of variance (ANOVA) results of the five variables contributing to the occurrence of abortion AE.

No.	Variables	Df	Sum square	Mean square	*F*-value	*P*-value (>*F*)
**Variable with statistically significant contribution to occurrence of abortion AE (*P*-value < 0.05)**	
1	Animal species	1	0.429	0.42856	24.7130	3.626E-05
2	Vaccination dose	16	1.214	0.07587	4.3749	4.361E-03
3	Vaccination route	3	1.17428	0.39143	22.5717	2.080E-07
**Variable without statistically significant contribution to occurrence of abortion AE (*P*-value > 0.05)**	
4	Animal age at vaccination	1	0.02042	0.02042	1.1774	0.2878442
5	Vaccine type	1	0.00100	0.00100	0.0578	0.8118476

### The Effects of Different Variables on Abortion AE in Vaccinated Animals

**FIGURE 6:**
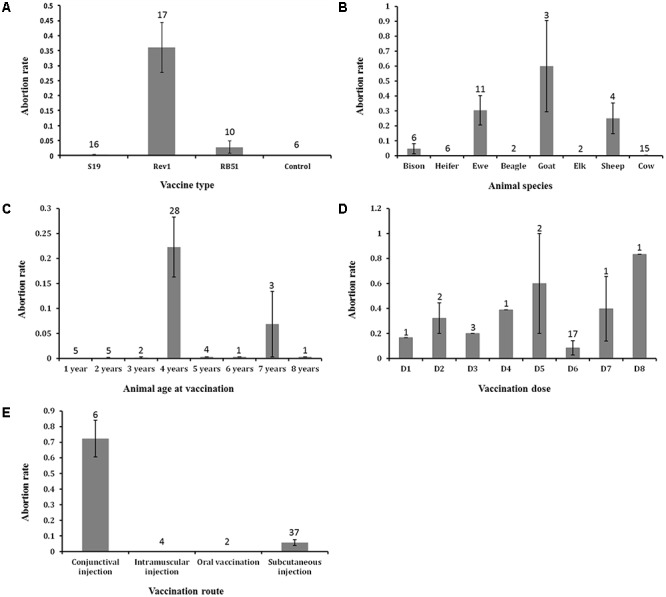
The effects of major five variables on abortion AE that occurred in vaccinated animals. **(A)** The effect of vaccine types on abortion AE. **(B)** The effect of animal species on abortion AE. **(C)** The effect of animal ages at vaccination on abortion AE. **(D)** The effect of vaccination doses on abortion AE. Abbreviations: D1, 1 × 10^5^; D2, 1 × 10^6^; D3, 7.5 × 10^6^; D4, 1 × 10^7^; D5, 1 × 10^8^; D6, 3 × 10^8^; D7, 1 × 10^9^; D8, 3 × 10^9^. **(E)** The effect of vaccination routes on abortion AE. Note that there is a statistically significant difference between the means of abortion rates of the conjunctival and subcutaneous injection groups (*P*-value < 0.05). In all the five bar charts, the error bars represent standard errors. The numbers in the plots indicate the numbers of experiment groups of vaccinated animals with the indicated variable settings.

As shown in **Figure 6**, we plotted out different variable parameters effect on the abortion AE. Although vaccine type as a variable is not statistically significantly correlated with abortion rate (**Table 3**), our analysis of the abortion rates associated with different vaccines from all annotated studies showed that Rev1 has the highest abortion rate of 36.1%, followed by RB51 (2.8%) and S19 (0.3%) (**Figure 6A**). Considering that since S19 is more virulent than RB51, it was a surprise that the average value of S19-associated abortion rate was less than the average of RB51-associated abortion rate. One reason for this is that the dose of RB51 vaccination is much larger than S19. A further *t*-test analysis showed that there was no statistically significant difference between these two groups (*P*-value > 0.1). These three vaccines were tested in eight different animal species for abortion occurrence (**Figure 6B**). The highest abortion rate was observed in the goat group, followed by ewe, sheep, bison, and cow. Based on our current annotated data, we have not found any abortion outcome associated with these three vaccines when they were vaccinated into heifer, beagle, and elk (**Figure 6B**). Most vaccine AEs were evaluated using 4-year-old animals, which also showed the highest abortion rate (22.2%) (**Figure 6C**). Eight different doses were used in vaccine animal studies (**Figure 6D**). The most commonly used dosage was 3 × 10^8^ CFU/animal, which appeared to be associated with relatively low average abortion rate compared to other dosages. Among four reported vaccination routes (**Figure 6E**), subcutaneous injection was the most commonly used method with 37 vaccination groups, and 5.7% of animals vaccinated with this route had the abortion AE. The highest abortion rate (72.3%) was observed in six groups of animals vaccinated with the conjunctival injection route. Interestingly, no animal abortion case was reported when the vaccination route was intramuscular injection or oral administration (**Figure 6E**).

## Discussion

The results of this study show that many AEs were associated with the three licensed brucellosis vaccines that were intentionally administered to domestic and wild ungulates as well as accidentally inoculated into humans. We collected all these animal or human AEs from the peer-review articles in PubMed database. The casual relationships between the occurrence of certain AE symptoms and the specific components of brucellosis vaccines could not be defined in this study. However, our collection and analysis of these AEs can help us improve the efficacy and safety of licensed brucellosis vaccines. To our best knowledge, this study represents the first reported ontology-based meta-analysis of adverse events associated with animal vaccines.

Most of human AEs are mild or moderate in severity (Spink et al., 1962; Ashford et al., 2004; Wallach et al., 2008). These AEs show flu-like symptoms (e.g., fever, chills, and headache) that are consistent with the clinical manifestations of human infection with *Brucella*. To date, no fatal AE or permanent damage has been reported to associate with brucellosis vaccines including S19, Rev1, and RB51 in humans. Some patients demonstrated symptoms of acute brucellosis after accidental exposure to brucellosis vaccines and necessitating hospitalization. The symptoms were gradually subsided and these patients were all recovered due to proper treatments or after time passed (Spink et al., 1962; Ashford et al., 2004). Since existing or potential AEs associated with humans after accidental vaccine exposure, safety measures (e.g., wear gloves and safety training) should be implemented in the persons who have opportunities to contact with these vaccines.

Although veterinary vaccines are being used with increasing frequency, the AEs associated with the vaccination of these vaccines are rarely reported. It is unclear whether this is due to infrequent occurrence of AEs, or under-recognition/under-reporting. When animals suffered AEs after vaccination, these AEs could not be reported like human vaccines AEs spontaneously reported by humans. At present, most of reported AEs associated with the use of animal vaccines were from the serologic evaluation and necropsy results for those vaccinated animals. Based on our study, in addition to abortion and arthropathy, no other AEs were reported to associate with S19 and/or Rev1 in animals. However, there are 19 AEs were reported after vaccination of RB51 in different animals (e.g., bison, deer mice, and buffalo). Our survey found that existing studies on AEs associated with S19 and/or Rev1 are not more than seven papers, while more than 16 peer-reviewed articles were focused on the safety of RB51 vaccination. Therefore, we cannot simply suggest that S19 and Rev1 are more secure than RB51 according to the number of reported AEs associated with them. In fact, RB51 is less virulent than S19 and Rev1 and show less chance to induce abortion or other serious adverse events. In addition, RB51 does not interfere with routine serological surveillance for brucellosis (Stevens et al., 1994). Nowadays, RB51 has become the preferred vaccine for cattle and likely a brucellosis vaccine for wildlife (Kreeger et al., 2002). Therefore, the reported more RB51-associated AEs are likely due to the less reports of AEs associated with the other vaccines.

Our AE meta-analysis identified that abortion is the only and most common AE associated with all the three licensed vaccines in animals. The further ANOVA statistics analysis indicated that animal species, vaccination route, and vaccination dose in the process of brucellosis vaccination are three important variables for determining the occurrence of abortion AE. Among those animals, the bovines (e.g., goat, ewe, and sheep) are the most frequently reported animals that suffered abortion after brucellosis vaccination (**Figure 6B**). This may be because the existing brucellosis vaccines are usually used to administer bovines, while they are not yet widely used for other animals.

For inoculation methods (**Figure 6E**), subcutaneous vaccination is the most commonly used vaccination route (Beckett and MacDiarmid, 1985; Elzer et al., 1998; Olsen and Holland, 2003; Ebrahimi et al., 2012; Singh et al., 2012). Conjunctival vaccination was associated with the highest abortion rate (Zundel et al., 1992). For oral and intramuscular vaccination used in brucellosis vaccines administration, no abortion case has been reported. In fact, at present, the oral or intramuscular vaccination method is rarely used to administer brucellosis vaccines to animals because of their economy and effectiveness need to be further improved, and related AE studies are scant.

For vaccination dose, a large spread of values was observed, ranging from a CFU of 1 × 10^4^ to a CFU of 3.4 × 10^10^. Depending on the dose administered during pregnancy, abortions will occur with variable frequency (**Figure 6D**). For example, when vaccinated Rev1 to the pregnant ewes with dosage of 1 × 10^9^ CFU, abortions occurred later at surprisingly severe rates (90.9%), while in the 1 × 10^8^ CFU ewes group, the abortion rate was only 20% (Zundel et al., 1992). To our best knowledge, there are no standard or foolproof vaccination dosages of the brucellosis vaccines use for pregnant or non-pregnant animals. However, a dose of 1–3.4 × 10^10^ CFU was recommended for vaccination of RB51 in calves (Stevens et al., 1997; Fosgate et al., 2003). Palmer et al. (1997) suggested that pregnant cattle can be safely vaccinated with 1 × 10^9^ CFU of RB51 without subsequent abortion or placentitis. Additionally, for S19, the dosage of 3 × 10^8^ CFU is the most commonly used for animal vaccination (Beckett and MacDiarmid, 1985; Herr et al., 1986; Palmer et al., 1997).

At present, with the exception of brucellosis vaccines, more and more veterinary vaccines are being used in animal industry. The United States Department of Agriculture has licensed more than 2,000 vaccines for use in animals (Arrioja-Dechert, 1999). Most of these vaccines are inactivated formulations, but more than 500 live vaccine formulations for animals are also licensed (Berkelman, 2003). However, there have been few studies focused on the AEs associated with these veterinary vaccines. Furthermore, the veterinary vaccines, especially many live attenuated vaccines were not tested for safety in humans. We do not know what AEs will occur when humans are inadvertently exposed to these vaccine products. Therefore, in future studies, we should invest more human and financial capital to report and analyze the veterinary vaccine adverse events. For example, like Vaccine Adverse Event Reporting System (VAERS) (Chen et al., 1994), some form of post-marketing surveillance system for AEs associated with the veterinary vaccines in animals and humans should be developed and implemented. These are crucial for us to monitor and improve the safety of existing vaccine products and design novel and more secure vaccines.

## Limitations

Some limitations in this study should be discussed. First, since the existing AEs associated with the three brucellosis vaccines are collected from the 27 peer-reviewed publications, and most of these researches belong to case-control studies, specific causal relationships between the collected AEs and the three brucellosis vaccines cannot be fully established. Second, there are currently big differences for the quantity of studies that focused on the safety of S19, Rev1, or RB51 vaccination. In the results section, we mentioned that our survey found only 6 and seven studies involved in AE case reports with S19 and Rev1, respectively. However, there are 16 papers committed to the security of RB51 and reported the corresponding AE results. Obviously, the quantity of studies on the security of a specific vaccine largely determines the variety and amount of its reported AEs. Therefore, the AE statistical results may not be reasonable quantitative criteria for the security and efficacy of the specific vaccine. Third, while the available literatures on adverse events in response to brucellosis vaccination are limited, there are some biases in our ANOVA results for the five variables contributed to abortion AE in vaccinated animals. For example, goats had highest abortion rate, but they only received the most ‘virulent’ vaccine (Rev1). In future studies, we should be performed additional level of analysis to account for multiple variables when more relevant data becomes available.

## Conclusion

While existing licensed brucellosis vaccines have dramatically reduced the incidence of many *Brucella* infections in animals and humans, these vaccines are able to induce many adverse events in vaccinated animals as well as accidentally inoculated humans. In this PubMed literature meta-analysis, we systematically collected and collated the AEs associated the existing three licensed brucellosis vaccines (i.e., S19, Rev1, and RB51). The OAE-based classification analysis showed that the animal AEs were mainly concentrated in immune and reproductive systems, while the human AEs usually involved in behavioral and neurological conditions. Furthermore, the ANOVA study indicated that three variables, including animal species, vaccination dose, and vaccination route are significantly associated with the occurrence of abortion AE in animals.

## Author Contributions

JX collected the data from PubMed database, added more than 20 AE terms to OAE, performed OAE-based meta-analysis, and wrote the first draft of the paper. JW collected and checked the original data. ZL, WW, and YP conceived general study design, and performed data analysis. YH provided the general project design, participated in OAE term review and updating, and supported data interpretation and paper writing. All authors edited and approved the manuscript.

## Conflict of Interest Statement

The authors declare that the research was conducted in the absence of any commercial or financial relationships that could be construed as a potential conflict of interest.

## Footnotes

1 http://www.ncbi.nlm.nih.gov/PubMed/

2 http://protege.stanford.edu/

3 https://cran.r-project.org/bin/windows/base/

## Supplementary Material

PRESENTATION S1 The *R* code for implementation of analysis of variance (ANOVA) for linear model fits (PDF).

PRESENTATION S2 The detailed retrieve information of 27 eligible peer-reviewed articles (PDF).

DATA SHEET S1 The original data curated from the 27 peer-reviews articles and the results of data transformation for ANOVA statistics analysis (XLSX).
