# Supplementary material for: Ontology-Based Meta-Analysis of Animal and Human Adverse Events Associated With Licensed Brucellosis Vaccines
Source: Front Pharmacol. 2018 May 15;9:503. doi: 10.3389/fphar.2018.00503 (PMC5962797; doi:10.3389/fphar.2018.00503)
Supplement: PRESENTATION S1 — The R code for implementation of analysis of variance (ANOVA) for linear model fits (PDF). [file Presentation_1.PDF]

Supplemental File 1. *R* code for implementation of ANOVA for linear model fits.

Below is the screenshot of R code and results:

```
> setwd(".")
> a<-read.table('data.txt', header=T, sep="\t");
> library(dprep)
> b<-disc.ew(a,c(5,6))
Warning message:
In if (out == "symb") { :
  the condition has length > 1 and only the first element will be used
> fit<-lm(Abortion_rate ~ ., data=b)
> anova(fit)
Analysis of Variance Table

Response: Abortion_rate
                            Df  Sum Sq Mean Sq F value    Pr(>F)    
Animal.species               1 0.42856 0.42856 24.7130 3.626e-05 ***
Animal.age.at.vaccination    1 0.02042 0.02042  1.1774 0.2878442    
Vaccine.type                 1 0.00100 0.00100  0.0578 0.8118476    
Vaccination.dose            16 1.21388 0.07587  4.3749 0.0004361 ***
Vaccination.route            3 1.17428 0.39143 22.5717 2.080e-07 ***
Residuals                   26 0.45088 0.01734                      
---
Signif. codes:  0 '***' 0.001 '**' 0.01 '*' 0.05 '.' 0.1 ' ' 1
> |
```

Screenshot Description:

In the above code, the data is provided in the data file called "data.txt". The data.txt content is obtained from the Supplemental File 3.

The *R* library of dprep is for data pre-processing and visualization functions for classification (https://cran.r-project.org/web/packages/dprep/index.html).

The disc.ew() function is a discretization method using the equal width method (https://www.rdocumentation.org/packages/dprep/versions/3.0.2/topics/disc.ew).

The linear model:

fit<-lm(Abortion_rate ~ ., data=b)

In this model, the variable "Abortion_rate" (*i.e.*, abortion rate) is the dependent variable, and all the other variables, represented as ".", are independent variables. This model tests how each of the independent variable affects the dependent variable "Abortion_rate".

The general R ANOVA for linear model fits method is explained here: https://stat.ethz.ch/R-manual/R-devel/library/stats/html/anova.lm.html.
