# Supplementary material for: Ontology-Based Meta-Analysis of Animal and Human Adverse Events Associated With Licensed Brucellosis Vaccines
Source: Front Pharmacol. 2018 May 15;9:503. doi: 10.3389/fphar.2018.00503 (PMC5962797; doi:10.3389/fphar.2018.00503)
Supplement: PRESENTATION S2 — The detailed retrieve information of 27 eligible peer-reviewed articles (PDF). [file Presentation_2.PDF]

**Supplemental File 2.** The list of 27 peer-reviewed articles [1-27] that are used in our meta-analysis.

[1] Blasco JM, Marin C, Debagues MPJ, Barberan M. Efficacy of Brucella-Suis Strain-2 Vaccine against Brucella-Ovis in Rams. Vaccine. 1993;11:1291-4.

[2] Olsen SC, McGill JL, Sacco RE, Hennager SG. Immune Responses of Bison and Efficacy after Booster Vaccination with Brucella abortus Strain RB51. Clin Vaccine Immunol. 2015;22:440-7.

[3] Diptee MD, Adesiyun AA, Asgarali Z, Campbell M, Adone R. Serologic responses, biosafety and clearance of four dosages of Brucella abortus strain RB51 in 6-10 months old water buffalo (Bubalus bubalis). Veterinary immunology and immunopathology. 2006;109:43-55.

[4] Olsen SC, Rhyan J, Gidlewski T, Goff J, Stoffregen WC. Safety of Brucella abortus strain RB51 in black bears. J Wildl Dis. 2004;40:429-33.

[5] Olsen SC, Holland SD. Safety of revaccination of pregnant bison with Brucella abortus strain RB51. J Wildl Dis. 2003;39:824-9.

[6] Kreeger TJ, DeLiberto TJ, Olsen SC, Edwards WH, Cook WE. Safety of Brucella abortus strain RB51 vaccine in non-target ungulates and coyotes. J Wildl Dis. 2002;38:552-7.

[7] Singh R, Basera SS, Tewari K, Yadav S, Joshi S, Singh B, et al. Safety and immunogenicity of Brucella abortus strain RB51 vaccine in cross bred cattle calves in India. Indian journal of experimental biology. 2012;50:239-42.

[8] Cook WE, Williams ES, Thorne ET, Taylor SK, Anderson S. Safety of Brucella abortus strain RB51 in deer mice. J Wildl Dis. 2001;37:621-5.

[9] Januszewski MC, Olsen SC, McLean RG, Clark L, Rhyan JC. Experimental infection of nontarget species of rodents and birds with Brucella abortus strain RB51 vaccine. J Wildl Dis. 2001;37:532-7.

[10] Cook WE, Williams ES, Thorne ET, Kreeger TJ, Stout GW, Schurig G, et al. Safety of Brucella abortus strain RB51 in bull elk. J Wildl Dis. 2000;36:484-8.

[11] Kreeger TJ, Miller MW, Wild MA, Elzer PH, Olsen SC. Safety and efficacy of Brucella abortus strain RB51 vaccine in captive pregnant elk. J Wildl Dis. 2000;36:477-83.

[12] Edmonds MD, Schurig GG, Samartino LE, Hoyt PG, Walker JV, Hagius SD, et al. Biosafety of Brucella abortus strain RB51 for vaccination of mature bulls and pregnant heifers. American journal of veterinary research. 1999;60:722-5.

[13] Elzer PH, Edmonds MD, Hagius SD, Walker JV, Gilsdorf MJ, Davis DS. Safety of Brucella abortus strain RB51 in Bison. J Wildl Dis. 1998;34:825-9.

[14] Palmer MV, Olsen SC, Cheville NF. Safety and immunogenicity of Brucella abortus strain RB51 vaccine in pregnant cattle. American journal of veterinary research. 1997;58:472-7.

[15] Zundel E, Verger JM, Grayon M, Michel R. Conjunctival vaccination of pregnant ewes and goats with Brucella melitensis Rev 1 vaccine: safety and serological responses. Annales de recherches veterinaires Annals of veterinary research. 1992;23:177-88.

[16] Crowther RW, Orphanides A, Polydorou K. Vaccination of adult sheep with reduced doses of Brucella melitensis strain Rev.1: Safety and serological responses. Tropical animal health and production. 1977;9:85-91.

[17] Palmer MV, Cheville NF. Effects of oral or intravenous inoculation with Brucella abortus strain RB51 vaccine in beagles. American journal of veterinary research. 1997;58:851-6.

[18] Beckett FW, MacDiarmid SC. The effect of reduced-dose Brucella abortus strain 19 vaccination in accredited dairy herds. The British veterinary journal. 1985;141:507-14.

[19] Corbel MJ, Stuart FA, Brewer RA, Jeffrey M, Bradley R. Arthropathy associated with Brucella abortus strain 19 vaccination in cattle. I. Examination of field cases. The British veterinary journal. 1989;145:337-46.

[20] Corbel MJ, Stuart FA, Brewer RA, Jeffrey M, Bradley R. Arthropathy associated with Brucella abortus strain 19 vaccination in cattle. II. Experimental studies. The British veterinary journal. 1989;145:347-55.

[21] Spink WW, Hall JW, 3rd, Finstad J, Mallet E. Immunization with viable Brucella organisms. Results of a safety test in humans. Bull World Health Organ. 1962;26:409-19.

[22] Ebrahimi M, Nejad RB, Alamian S, Mokhberalsafa L, Abedini F, Ghaderi R, et al. Safety and efficacy of reduced doses of Brucella melitensis strain Rev. 1 vaccine in pregnant Iranian fat-tailed ewes. Veterinaria italiana. 2012;48:405-12.

[23] Blasco JM, Diaz R. Brucella melitensis Rev-1 vaccine as a cause of human brucellosis. Lancet. 1993;342:805.

[24] Olle-Goig JE, Canela-Soler J. An outbreak of Brucella melitensis infection by airborne transmission among laboratory workers. Am J Public Health. 1987;77:335-8.

[25] Palmer MV, Olsen SC, Gilsdorf MJ, Philo LM, Clarke PR, Cheville NF. Abortion and placentitis in pregnant bison (Bison bison) induced by the vaccine candidate, Brucella abortus strain RB51. American journal of veterinary research. 1996;57:1604-7.

[26] Wallach JC, Ferrero MC, Victoria Delpino M, Fossati CA, Baldi PC. Occupational infection due to Brucella abortus S19 among workers involved in vaccine production in Argentina. Clinical microbiology and infection : the official publication of the European Society of Clinical Microbiology and Infectious Diseases. 2008;14:805-7.

[27] Ashford DA, di Pietra J, Lingappa J, Woods C, Noll H, Neville B, et al. Adverse events in humans associated with accidental exposure to the livestock brucellosis vaccine RB51. Vaccine. 2004;22:3435-9.
